# Developing a quantum computing model for sequence annotation of interferon protein

**DOI:** 10.1016/j.csbj.2025.11.027

**Published:** 2025-11-14

**Authors:** Bing Li, Yangchao Yu, Fubo Ma, ZiYang Dong, Le Zhang

**Affiliations:** a College of Computer Science, Sichuan University, Chengdu, China; b West China Biomedical Big Data Center, West China Hospital, Sichuan University, Chengdu, China

**Keywords:** Quantum computing, Sequence annotation, Interferon, DNA sequencing

## Abstract

Interferon-related research has important implications for understanding how biological immune systems function. With the development of DNA sequencing in recent years, interferon has been found in an increasing number of species, and many genome sequences remain to be annotated. Since BLAST is a commonly used sequence comparison method and conventional methods have difficulty to optimize the time complexity of the BLAST algorithm, quantum computing sheds light on accelerating BLAST for sequence annotation after DNA sequencing. Moreover, owing to the limitations of quantum devices, related quantum circuit optimization methods are needed to optimize the circuits of quantum algorithms to reduce the qubits required and complexity of the circuits.

This work presents a quantum computing model for sequence annotation after DNA sequencing. First, we propose a quantum gene sequence alignment algorithm that optimizes the time complexity of the second step of BLAST from linear to semilinear by combining Grover's quantum search algorithm and the quantum 01 string alignment algorithm. Second, we propose a quantum circuit optimization method based on a truth table that successfully reduces the number of quantum resources needed to implement the quantum algorithm. Third, we propose a visualization and analysis website that provides online circuit generation and simple experiment running. The experimental results show that our proposed methods can be successfully performed both on virtual and real quantum computer, verifying the usability of the algorithm.

Website: http://www.combio-lezhang.online/QGSA.

## Graphical abstract

**Figure:**
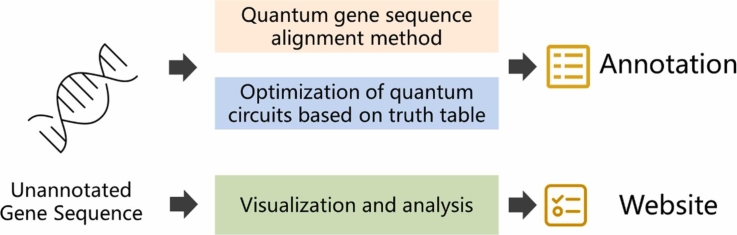

## 1 Introduction

Interferon is a kind of host-specific cell signalling protein that is usually secreted by animal cells after they are infected by specific viruses to activate the immune system. The generation and treatment of many diseases have been related to interferon in recent years, such as various types of cancer [1], [2], diabetes [3], rheumatism [4], and transplantation rejection [5]. Researches on interferon have important implications for understanding how biological immune systems function. Given the important role of interferon in immunoreactions, related researches can not only greatly promote the development of immunology but also benefit vaccine research, disease mechanism analysis and personalized medical diagnosis.

With the development of DNA sequencing and whole-genome sequencing (WGS) in recent years [6], [7], [8], [9], interferon has been found in an increasing number of species. As an important component of vertebrates, amniotes are highly valuable for interferon research. Amniotic animals refer to vertebrates with amniotic membranes during development [10], [11], such as birds, reptiles, and mammals. At present, most studies on interferon in amniotic animals are based on WGS [12], [13], [14] and the NCBI interferon protein sequence annotation process [15], [16], the cores of which are sequence alignment methods and protein sequence prediction models.

BLAST is the most commonly used method among sequence comparison methods. Therefore, many optimizations for BLAST have been proposed, such as pruning in the process [17] and parallel computing on multicore CPUs [18] or GPUs [19], [20]. These methods have increased the efficiency of BLAST, but the calculation method of BLAST has not been fundamentally changed. The time complexity of the optimization algorithm is still constant. In recent years, with the rapid development of quantum computing [21], [22], several new quantum string matching algorithms based on the Grover algorithm [23], [24] have been proposed [25], shedding light on accelerating the BLAST method. Therefore, how to construct such a quantum gene sequence alignment method that can optimize the time complexity of BLAST in the interferon sequence alignment scenario by a quantum computing algorithm has become our first scientific problem.

It is challenging for us to deploy and execute quantum algorithms on real quantum computers, since current quantum computers are usually noisy intermediate-scale quantum (NISQ) computers [26], [27], [28], which have defects such as limited usable qubits and possible errors introduced by quantum gates in the circuit. Since complex quantum circuits introduce large errors and produce unreliable experimental results, scientists have developed a series of quantum circuit optimization algorithms [29], [30], including gate-level optimization [31], [32], graph-based optimization [33], [34], and other algorithmic optimization [35], [36], to decrease the hardware requirements to execute quantum circuits. The graph-based ZX-calculus method [37], [38], [39] can deduce complex quantum circuit transformations by simple graph transformation and combination to optimize various quantum circuits. However, the ZX-calculus method is time-consuming because it generalizes all the quantum circuits that can be optimized. In the circuits proposed by our quantum gene sequence alignment method, the number of qubits is proportional to the length of the whole genome sequence [13], which is essentially hundreds of millions for a single organism, resulting in incredible time overhead if the ZX-calculus method is used to optimize quantum gene sequence alignment circuits. Therefore, how to construct an efficient quantum circuit optimization algorithm for the quantum gene sequence alignment method and effectively decrease the required quantum computing resources becomes our second scientific problem.

Previous studies related to quantum computing have focused mainly on the theoretical analysis and derivation of quantum algorithms, which usually lack intuitive examples, implementation methods, and visualization tools for understanding quantum algorithms [40]. In terms of the visualization of quantum computing, there are several tools for quantum circuit construction and circuit visualization, such as QuaCiDe [41] and Qiskit [42]. However, these tools provide interfaces to only usable quantum gates and still need specific programming to demonstrate the quantum circuits for a particular algorithm. Therefore, how to develop a visualization and analysis website for our quantum gene sequence alignment method and quantum circuit optimization algorithm based on web visualization technology with available online tools has become our third scientific problem.

On the basis of these three scientific problems, we propose corresponding innovations. First, we construct a quantum sequence alignment algorithm by implementing quantum string matching based on the Grover algorithm, which can greatly decrease the operational complexity and replace the second step of the BLAST algorithm. Second, we propose a quantum circuit optimization algorithm based on a truth table, which can optimize the quantum circuit of the quantum sequence alignment algorithm and decrease the time complexity of quantum circuit optimization and the space complexity of the quantum sequence alignment algorithm. Finally, we propose a visualization and analysis website that provides online circuit generation and simple experiment running.

In conclusion, a quantum computing model for sequence annotation after DNA sequencing is proposed for our study, which develops the quantum gene sequence alignment method to optimize the time complexity of BLAST and builds up a quantum circuit optimization algorithm to decrease the requested quantum resources. We also developed an online visualization and analysis website that provides online circuit generation and simple experiment running. As a result, virtual and real quantum computer experiments demonstrate the successful deployment of our quantum gene sequence alignment. Moreover, the circuits are simplified, and the qubits requested are reduced after circuit optimization so that examples are successfully executed on a real quantum computer. Finally, we have discussed the current obstacles and future potential of employing the quantum algorithm for sequence alignment and relevant DNA sequencing applications in the discussion part.

## 2 Materials and methods

### 2.1 Quantum gene sequence alignment method

**Fig. 1:**
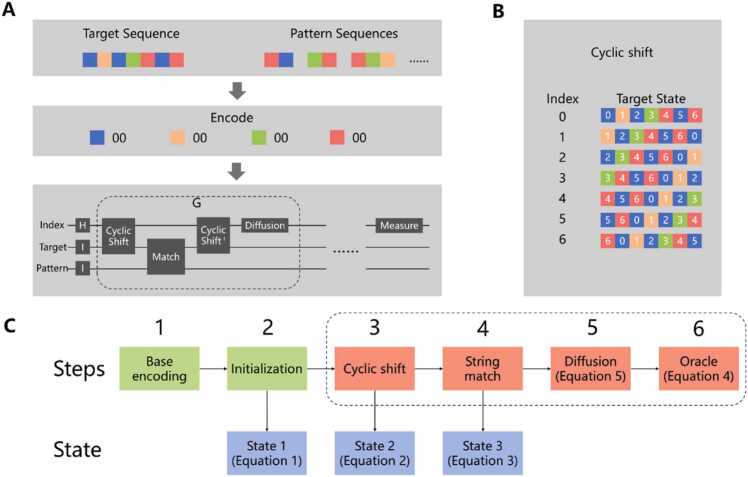
Overview of the quantum gene sequence alignment method. (A) Circuit of the quantum gene sequence alignment method. (B) Illustration of the cyclic shift.

The BLAST process is composed of three steps [43]. In the first step, the words with scores greater than the threshold are screened out according to a score transfer matrix such as PAM-120. In the second step, the selected words are employed to search the index in the database to find the matching site, where the database index records the position of all base sequences corresponding to the specified word size in the whole genome sequence. The third step takes the matching site in the second step as the centre, expands outwards through the MSP algorithm [44], and finally finds the locally optimal match. Since our goal is to employ a quantum algorithm to optimize the time complexity for the second step from linear to sublinear, we combined Grover's quantum search algorithm [40] and the quantum 01 string alignment algorithm [25] to construct a quantum gene sequence alignment (QGSA) algorithm with specific data characteristics and scene requirements (Fig. 1).

The study of interferon involves two main sequences: one is the target sequence, and the other is the pattern sequence. The target sequence refers to the sequence of unknown function, while the pattern sequence is a known sequence used to compare with the target sequence. The pattern sequence is usually a well-studied standard sequence that represents the characteristics of a certain class of sequences. In the gene sequence alignment of interferon proteins, the unannotated whole genome sequence is the target sequence whose length is represented byN. Known interferon protein sequences are pattern sequences whose length is represented by M.

We present the QGSA algorithm in the following steps and illustrate it in Fig. 1C.

First, the QGSA algorithm encodes gene sequences. Before sequence alignment, binary coding of the four ACGT bases in the target sequence and pattern sequence is carried out, as shown in Table 1. The lengths of the encoded target sequence and encoded pattern sequence are 2N and 2M, respectively.

**Table 1:** Base encoding method.

A	C	G	T
00	01	10	11

Second, the initial state is established. A total of 3 types of qubits is needed: index qubits, target string qubits, and pattern string qubits. The index qubits use the H gate to construct the superposition state in the initialization stage, as well as the target string qubits and pattern string qubits use the X gate to construct the same state as the target and pattern string construct in the initialization stage. The state is shown in Eq. 1. Here, $|\left(t_{i}\right\rangle$ is the $i_{\mathrm{th}}$ qubit of the target string, and $|p_{j}\rangle$ is the $j_{\mathrm{th}}$ qubit of the pattern string. 2N and 2M are the lengths of the encoded target string and pattern string, respectively. Notably, for subsequent representations, let us assume that 2N is a power of 2; that is, 2$N=2^{n}$. We discuss later the situation in which 2N is not a power of 2.

(1) $$\begin{array}{c} \frac{1}{\sqrt{2N}}\sum_{r=0}^{2N-1}|\left(r\right\rangle \left[\underset{i=0}{\overset{2N-1}{⨂}}|\left(t_{i}\right\rangle \right]\left[\underset{j=0}{\overset{2M-1}{⨂}}|p_{j}\rangle \right]\# \end{array}$$

Third, cyclic shift to the state is performed. We take the index qubits as the control bit and apply CSWAP gates to the target string qubits to carry out the cyclic shift and realize the effect that turns the target string qubits into a multistate superposition. After this step, the state is shown by Eq. 2. This step is the core of the quantum gene sequence alignment algorithm, which changes the state of the target qubits according to the state of the index qubits. Here, the target string ACGT is taken as an example to illustrate by Table 2, which shows how the state of the target qubits changes according to the state of the index qubits. The first target qubit is shown in red in the table. The cycle step in the example is 1; thus, each cycle shifts 1 qubit to the left.

(2) $$\begin{array}{c} \frac{1}{\sqrt{2N}}\sum_{r=0}^{2N-1}|\left(r\right\rangle \left[\underset{i=0}{\overset{2N-1}{⨂}}|\left(t_{i+r}\right\rangle \right]\left[\underset{j=0}{\overset{2M-1}{⨂}}|p_{j}\rangle \right]\# \end{array}$$

**Table 2:** Truth table for the cyclic shift.

index qubits	target qubits
|000〉	|00011011〉
|001〉	|00110110〉
|010〉	|01101100〉
|011〉	|11011000〉
|100〉	|10110001〉
|101〉	|01100011〉
|110〉	|11000110〉
|111〉	|10001101〉

Fourth, the shifted target string and pattern string are matched. After the cyclic shift, the CNOT gate is used to detect whether the first 2M qubits of the target and pattern string are matched. The state after the CNOT gate is applied as follows:

(3) $$\begin{array}{c} \frac{1}{\sqrt{2N}}\sum_{r=0}^{2N-1}|\left(r\right\rangle \left[\underset{i=0}{\overset{2N-1}{⨂}}|\left(t_{i+r}\right\rangle \right]\left[\underset{j=0}{\overset{2M-1}{⨂}}|p_{j}⨁t_{j+r}\rangle \right]\# \end{array}$$

Fifth, the phase of the correct matching state is reversed, thereby increasing its amplitude. The state of the pattern qubits in Eq. 3 indicates the number of mismatches in the first 2M qubits of the target and pattern string. If and only if there is a perfect match are the states of the pattern sequence qubits all $\left(|0\right\rangle$; then, the phase of this matched state is reversed using $U_{f}$, as shown in Eq. 4. The phase inversion operation $U_{f}$ is physically completed by the X gate and MCZ (multiple-control Z) gate. We implement the Oracle operator for our problem by performing the above phase inversion operations.

(4) $$\begin{array}{c} U_{f}\left(|p_{0}p_{1}\ldots p_{2M-1}\right\rangle =\left\{\begin{array}{c} -\left(|p_{0}p_{1}\ldots p_{2M-1}\right\rangle \;\sum_{i=0}^{2M-1}p_{i}=0\\ +\left(|p_{0}p_{1}\ldots p_{2M-1}\right\rangle \;\sum_{i=0}^{2M-1}p_{i}\neq 0 \end{array}\right)\# \end{array}$$

Sixth, the diffusion operator is performed. We apply the diffusion operator in the Grover algorithm, which aims to form a reflection about $|\left(r\right\rangle$. To construct the circuit, this operation can be decomposed into a reflection about ${\left(|0\right\rangle }^{⨂n}$, which can be performed by a multiple-control Z (MCZ) gate after $H^{⨂n}$ gates (shown in Eq. 5).

(5.1) $$\begin{array}{c} D=2|\varphi \rangle \langle \varphi |-I=H^{⨂n}\;(2{\left(|0\right\rangle }^{⨂n}{\left\langle 0\right|}^{⨂n}-I)\;H^{⨂n}\# \end{array}$$

(5.2) $$\begin{array}{c} \left|\varphi \right\rangle =\frac{1}{\sqrt{2N}}\sum_{r=0}^{2N-1}|\left(r\right\rangle \# \end{array}$$

Finally, the Grover algorithm is applied several times, and the index qubits are subsequently observed to obtain the matching position of the pattern string in the target string.

Similar to the Grover algorithm, when the length of the target sequence is N and the length of the pattern sequence is M. We need no more than $\sqrt{N}$ repetitions to increase the amplitude of the correct matches. Specifically, the necessary number of repetitions can be reduced to $\lfloor \pi /4\;\sqrt{\left(N/M\right)}+1\rfloor$ [45].

Notably, simply replacing the base with the encoded 01 string is not enough to solve the problem. For example, string 0011 corresponding to AT will generate 1001 (GC) and 0110 (CG) after cyclic shift of 1 and 3 qubits, respectively, resulting in characters that do not exist in the original string; thus, it is necessary to adjust the shift interval. For cyclic shift of $2^{q}$ qubits in a target string with $k\times \;2^{n}$ qubits (using k qubits to represent a character), the interval for the shift should be $k\times \;2^{q}$, $k\times \;2^{q+1}$ …… $k\times \;2^{n-1}$.

In addition, some minor problems in the 01-string alignment algorithm exist; for example, the cyclic shift cannot be realized when the target string length is not a power of 2, and when too many qubits are subjected to the cyclic shift (greater than N−M), start−end contact occurs in the first M bits, resulting in a substring that does not exist. Taking the alignment between 1001 and 11 as an example, after the cyclic shift of 3 qubits, the target string becomes 1100, which matches 11 but not the desired result. Therefore, a better choice is to use 3 qubits to represent a base, and an additional 100 is introduced to represent the termination character $. The terminating character does not match any of the base pairs; thus, adding the terminating character at the end of the target string can solve the above problem. Moreover, when the length of the target string is not a power of 2, we can fill it with the terminating character, which does not have any side effects on the result.

### 2.2 Optimization of quantum circuits based on a truth table

Owing to the hardware defects of quantum computers, the actual execution effect of quantum circuits is limited by the number of qubits and the noise of physical quantum computers. Thus, quantum circuits must be optimized to reduce the hardware requirements of execution. ZX-calculus is a very helpful tool and method for analysing and reducing the complexity of circuits [38]. The complex optimization process of this method is applicable to all quantum circuits, and its time complexity is $O(s^{2}N^{2})$ [38]. Here, s represents the number of nodes in the ZX diagram, and N represents the number of qubits.

Owing to its high time overhead, it is difficult to apply ZX-calculus to optimize the QGSA method. Moreover, the space complexity of QGSA algorithm is too high to be employed for the long sequences. For those reasons, we propose the optimization of quantum circuits based on the truth table (OPTIQUE) algorithm. Since the OPTIQUE algorithm records the evolution of quantum states on the basis of the characteristics of quantum superposition states generated by the cyclic shift of the QGSA algorithm with respect to the truth table, it can reduce the physical resources needed to construct QGSA circuits with low time complexity.

The OPTIQUE algorithm is as follows: In the QGSA algorithm, only 2 M target bits participate in the subsequent operation to obtain the results. On the basis of the original quantum circuit, we can remove the target qubits that do not participate in the subsequent operation and construct a simplified truth table. Because of the quantum superposition state of the index bits, applying a quantum gate to one index bit affects the corresponding state of the other qubits; thus, we must reconstruct the quantum circuit. In this process, the demand for the number of qubits in the quantum circuit is reduced because we removed the unused target qubits.

The core of the OPTIQUE algorithm is an optimization subroutine, which takes the truth table of the cyclic shift, the $i_{\mathrm{th}}$ target qubit $t_{i-1}$, the original quantum circuit of the index, and the $i_{\mathrm{th}}$ target qubits as the input and outputs of the optimized circuit of the index and the $i_{\mathrm{th}}$ target qubits. The steps are as follows and in order to present the algorithm clearer, an intuitive example is presented in the supplementary material Method S2.

First, we obtain a simplified truth table by removing the unused qubits from the truth table of the cyclic shift and extract ${\mathrm{st}}_{i-1}$, which is the state matching in $t_{i-1}$ and the index qubits, from the simplified truth table.

Second, we constructed counting tables. For each target qubit, we initialize a counting table with dimensions N×logN, which is filled with a value of 0.

Third, we iterate through the index and add pointers. During the iteration of index i, we rewrite i into binary, set the number of 1 s in this binary representation to j, and set the positions of 1 s in this binary to $O_{i}$. We add a pointer from column j and row i to column j of row k, where k meets the requirements in Eq. 6. Here, $O_{k}$ represents the set of positions of 1 s in the binary of k.

(6) $$\begin{array}{c} k\;s.t.\;i<k\mathrm{and}O_{i}\in O_{k}\# \end{array}$$

Fourth, we iterate through the index and update the counting table. For each target qubit, $t_{\mathrm{ki}}$ represents the corresponding state value of the target qubit $t_{k}$ in ${\mathrm{st}}_{k-1}$ when index=i, which is called the target qubit value. If index=0 and $t_{k0}=1$, we apply the X gate on the quantum circuit corresponding to the target qubit $t_{k}$. If index≠0, we calculate the sum of all the columns in row index+1 of the counting table. If the sum is an even number, we set $\mathrm{temp}=t_{k0}$; otherwise, $\mathrm{temp}=\overset{®}{t_{k0}}$ (here, temp is a temporary data variable). Then, we compare temp with $t_{\mathrm{kindex}}$. If $\mathrm{temp}\neq t_{\mathrm{kindex}}$, we add the quantum gate according to the index string. This step involves two rules:

Rule 1: We rewrite index into binary $\mathrm{IQ}S_{\mathrm{index}}$. If only one position of $\mathrm{IQ}S_{\mathrm{index}}$ is 1, the index qubit corresponding to this position is used as the control qubit, and $t_{k}$ is used as the target qubit. If $\mathrm{IQ}S_{\mathrm{index}}$ has more than one position of 1 s, the index qubit corresponding to each position of 1 is used as the control qubit, and $t_{k}$ is used as the target qubit to apply the Toffoli gate.

Rule 2: For each added quantum gate (except the X gate), we must update its counting table. For example, a quantum gate is added when iterating index i, and the position of number 1 in binary $\mathrm{IQ}S_{i}$ is j. Then, we need to add one to all the positions indicated by the pointer starting from column j and row i+1 of the counting table.

The OPTIQUE algorithm is based on the above optimization subroutines. Each time the optimization subroutine runs, it can optimize part of the quantum circuit. If the optimization subroutine is aimed at the $i_{\mathrm{th}}$ target qubit $t_{i-1}$, then the $i_{\mathrm{th}}$ target qubit quantum circuit is optimized after the optimization subroutine is finished. After running a total of 2 M times, the quantum circuit of the index qubit and the target qubit can be connected, thus optimizing the quantum circuit of the index qubit and the target qubit.

After that, the quantum circuit of the pattern qubit is built independently from the other circuits. We can add an X gate to the quantum circuit of the pattern qubit, and then connect it with the target qubit quantum circuit. 2M CNOT gates are requested for the connection of the quantum circuit. The control qubit of the $i_{\mathrm{th}}$ CNOT gate is the $i_{\mathrm{th}}$ target qubit $t_{i-1}$, and the target qubit is the $i_{\mathrm{th}}$ pattern qubit $p_{i-1}$.

### 2.3 Visualization and analysis websites

**Fig. 2:**
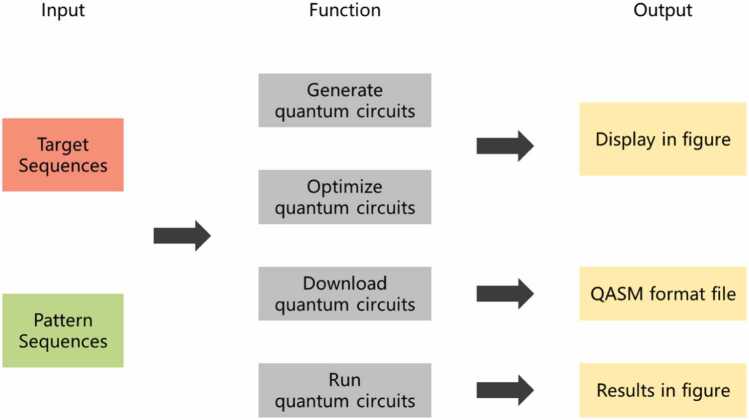
Architecture and function of the visualization and analysis website.

We provide a visualization and analysis website that enables the presentation of the QGSA and OPTIQUE algorithms, the main architecture and function of which are shown in Fig. 2. The website has the following main functions: generate circuits, optimize circuits, download circuits and run the circuits. Detailed information about the websites can be found in the Supplementary Material M3.

## 3 Results

### 3.1 Quantum experiments for QGSA

**Fig. 3:**
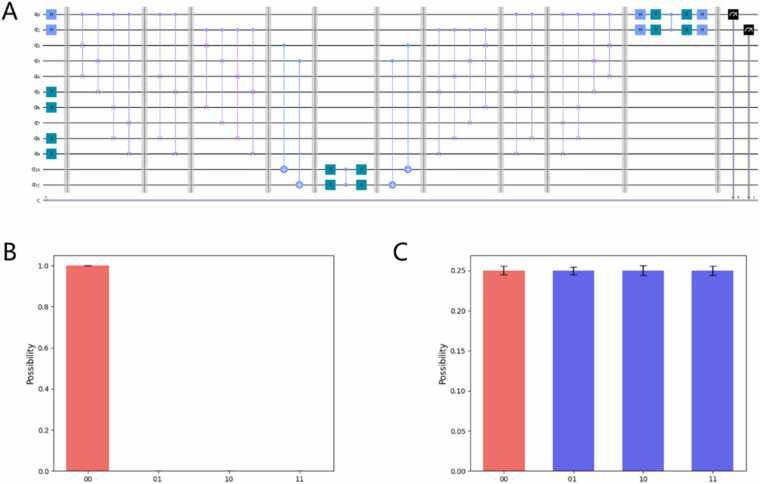
QGSA results. (A) Quantum circuit for pattern string A and target string ACGT. (B) Observation results of the virtual quantum computer experiment. (C) Observation results of the real quantum computer experiment.

To answer the first scientific question, we propose a quantum circuit for the QGSA algorithm, which is detailed in Section 2.1. In this experiment, pattern string A and target string ACGT are taken as examples, whose quantum circuits are shown in Fig. 3A. To validate the circuit, we conducted virtual quantum computer experiments and real quantum computer experiments, respectively. More details about the experiments can be found in the Supplementary Material.

On the one hand, Fig. 3B shows the results of the virtual quantum computer experiment. Since the objective of the experiment is to find pattern string A, we expect the observed result to be ‘00’, which is the code corresponding to A. Since the result is consistent with our expectation (Fig. 3B), the correction to our circuit of the quantum gene sequence alignment algorithm is validated.

On the other hand, Fig. 3C shows the results of the real quantum computer experiment. The experimental results (Supplementary Table S1) reveal that the differences between the observed probabilities of target state “00” and other nontarget states are nonsignificant because of insufficient overall accuracy. This result is caused by the excessive errors introduced by the large number of gates because several quantum operations must be divided into combinations of several basic quantum gates, and additional SWAP operations are needed due to the limitations of real quantum computers. Thus, Fig. 3C and above analysing indicate that we need optimize the quantum circuits to reduce the excessive errors introduced by the complex circuits.

In addition to the experimental results, we analyse the time complexity of the algorithm. The time complexity of the QGSA algorithm is related to the time complexity of the Grover algorithm and the Oracle operator. The target sequence with a length ofN is considered. The classical Grover algorithm without considering the Oracle operators has a time complexity of $O(\sqrt{N})$, which is the number of iterations of the algorithm. Next, we compute the time complexity of the QGSA algorithm by multiplying the time complexity of the Oracle operator by the time complexity of the Grover algorithm.

In the Oracle operator, the comparison steps using the CNOT gate can be parallel computed, and the time complexity is constant. Therefore, the time complexity of the cyclic shift is the key point, which is reflected in both the index qubit and the target qubit. During the cyclic shift, the target qubit can reach the expected position by exchanging $\log_{2}N$ times at most; thus, the time complexity of the target qubit is $O(\log_{2}N)$.

Since the number of index qubits is small ($\log_{2}N$), the complexity is clearly at least O(N) in the case of $N\log_{2}N$ exchanges if the controlled swap operation is carried out directly. However, by fan-out through the CNOT gates [25], the number of index bits can be increased to N by fan-out $\log_{2}N$ times, and then a cyclic shift can be achieved by controlling the swap of layer $\log_{2}N$. Therefore, the number of layers requested for a single index qubit is reduced to ${2\log}_{2}N$, and we can simultaneously compute $\log_{2}N$ index qubits; thus, the time complexity of the Oracle operator is reduced to $O({\log_{2}N}^{2})$. Finally, the overall complexity of the QGSA algorithm is reduced to $O(\sqrt{N}{\log_{2}N}^{2})$.

For comparison, the time complexity for the second step of BLAST is O(N) [43]. As we stated above, many methods for optimizing BLAST, such as pruning [17] and parallel computing on multicore CPUs [18] or GPUs [19], [20] have been proposed. These methods have increased the efficiency of BLAST, but the time complexity of BLAST remains O(N). Therefore, in contrast, our proposed QGSA method successfully optimizes the time complexity for the second step from linear $O\left(N\right)\;$to $O(\sqrt{N}{\log_{2}N}^{2})$.

### 3.2 Experimental results for the OPTIQUE algorithm

To answer the second scientific question, we propose an optimization algorithm for quantum circuits of the QGSA algorithm based on a truth table, which is detailed in Section 2.2.

**Fig. 4:**
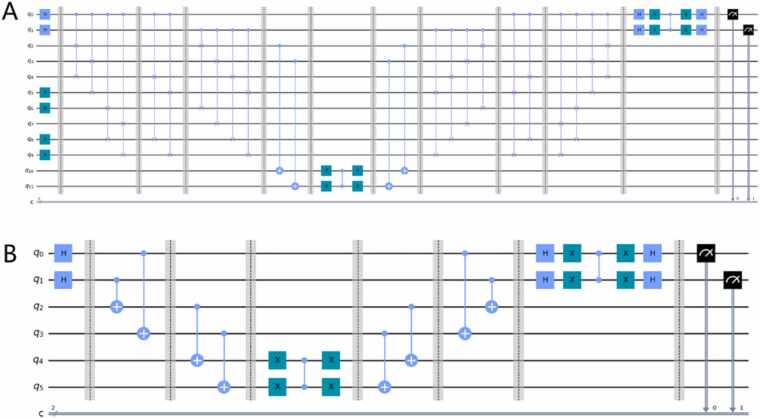
Comparison of quantum circuits before and after optimization. (A) Quantum circuits before optimization. (B) Quantum circuits after optimization.

Taking pattern string A and target string ACGT as an example, a comparison of the quantum circuits before and after optimization is shown in Fig. 4. The quantum circuits before optimization are shown in Fig. 4A, and the quantum circuits after optimization are shown in Fig. 4B. The optimized quantum circuit uses fewer qubits (6 qubits) than the original circuit does (12 qubits), and the number of quantum gates used by the quantum circuit in the example is reduced from 42 to 22.

To further verify the effectiveness of our proposed OPTIQUE method, we construct four separate experiments by looking for pattern strings A, T, C, and G in the target sequence ACGT and conduct both virtual quantum computer experiments and real quantum computer experiments on the quantum circuits before and after optimization for each case. More details about the experiments can be found in the Supplementary Material.

**Fig. 5:**
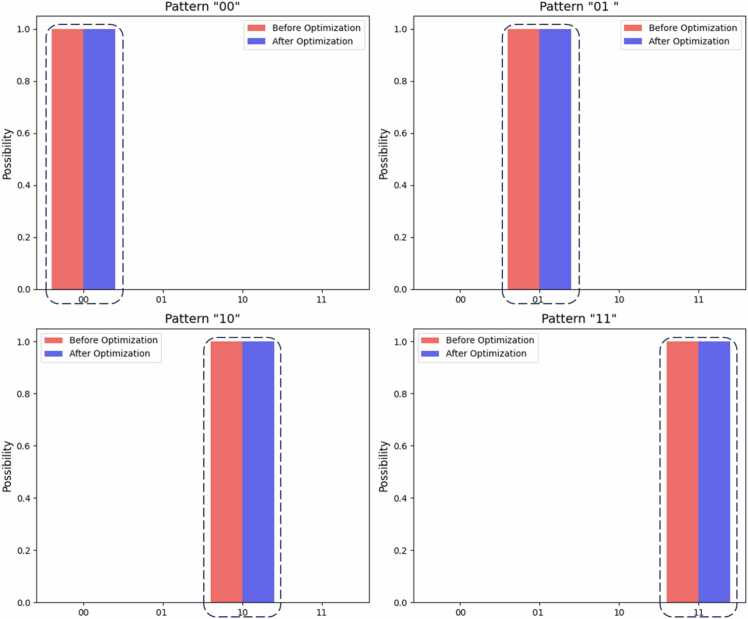
Observation results of the virtual quantum computer experiment.

On the one hand, Fig. 5 shows the observation results of the virtual quantum computer experiment. As shown in Fig. 5, the quantum circuits before and after optimization yielded the same results in the four experiments, and the correct observation results were obtained. The correct observation results refer to the observed state with a probability of 1, which is the state of the pattern string that we expect to search. The results for the virtual quantum computer prove that the circuits before and after optimization are equivalent.

**Fig. 6:**
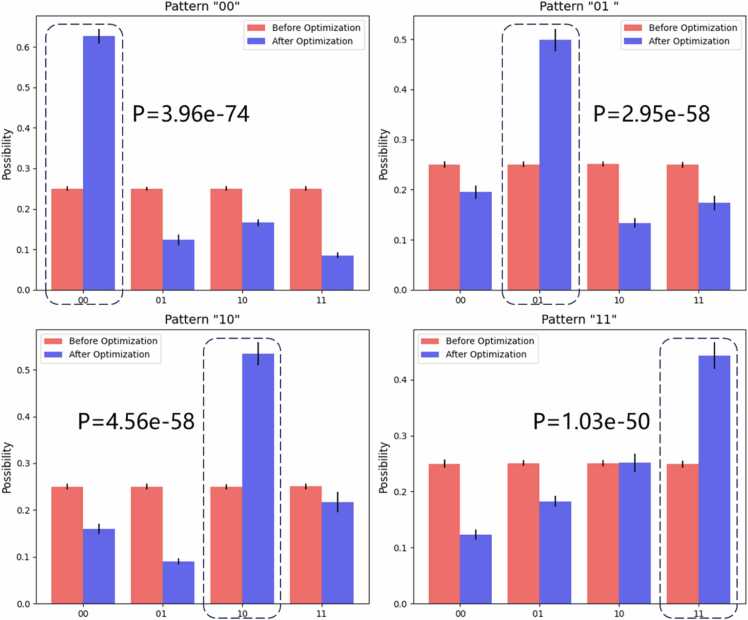
Observation results of the real quantum computer experiment. The P value in the figure was calculated using a T-test.

On the other hand, Fig. 6 shows the observation results from the real quantum computer experiment. As shown in Fig. 6, we compare the observed results of the quantum circuits before and after optimization, and the correct positions that we expect to observe are indicated by the dotted box in the figure. In addition, we perform a T-test [46], [47], [48], [49] using the probability of the correct position before and after optimization to test the differences. The data in Fig. 6 demonstrate that the probability of finding the correct position after optimization is significantly greater than that before optimization. The results prove that the error of the results is greatly reduced after optimization, and the probability of observing the correct position is obviously greater than that before optimization, which validates the effectiveness of our proposed OPTIQUE algorithm.

In addition to the experimental results, we analyse the time complexity of the algorithm. The target sequence with a length ofN and pattern sequences with a length of M are considered. The time complexity of OPTIQUE consists of two parts as follows. The time complexity of the optimization subroutine is O(N), and the time complexity of the adding and connecting pattern qubit circuits is O(M). Although we must run the optimization subroutine 2M times, each run of the optimization subroutine is independent and can be implemented in parallel. Thus, the time complexity is still O(N). In this study, since the target string is much longer than the pattern string is, we can consider that the time complexity of the OPTIQUE method is O(N).

In addition, since the OPTIQUE method removes qubits that do not participate in subsequent operations in the optimization subroutine, we can make the qubits that encode the target string the same as those of the pattern string, which effectively reduces the demand for the number of qubits. The space complexity of QGSA algorithm before optimization is $O(N+M+\log_{2}N)$. Afterwards, the space complexity of the circuit optimized by the OPTIQUE method becomes $O(M+\log_{2}N)$.

To further demonstrate the usability of our algorithm and explore its applicable scope under the current hardware conditions, we apply our algorithm on the long sequences. First of all, we have to note whether using classical computer simulations or real quantum computers, the sequences that can be processed at present have not yet reached a practical scale, but benefit from our OPTIQUE algorithm, we can process much longer sequences with fewer qubits.

Here, we take the interferon regulatory factor 2 binding protein 1 of the Iconisemion striatum (GenBank: SBP18523.1) and a part of contig APS_c27392_g4_i2 (GenBank: HADW01017123.1) as the experimental subjects.

**Fig. 7:**
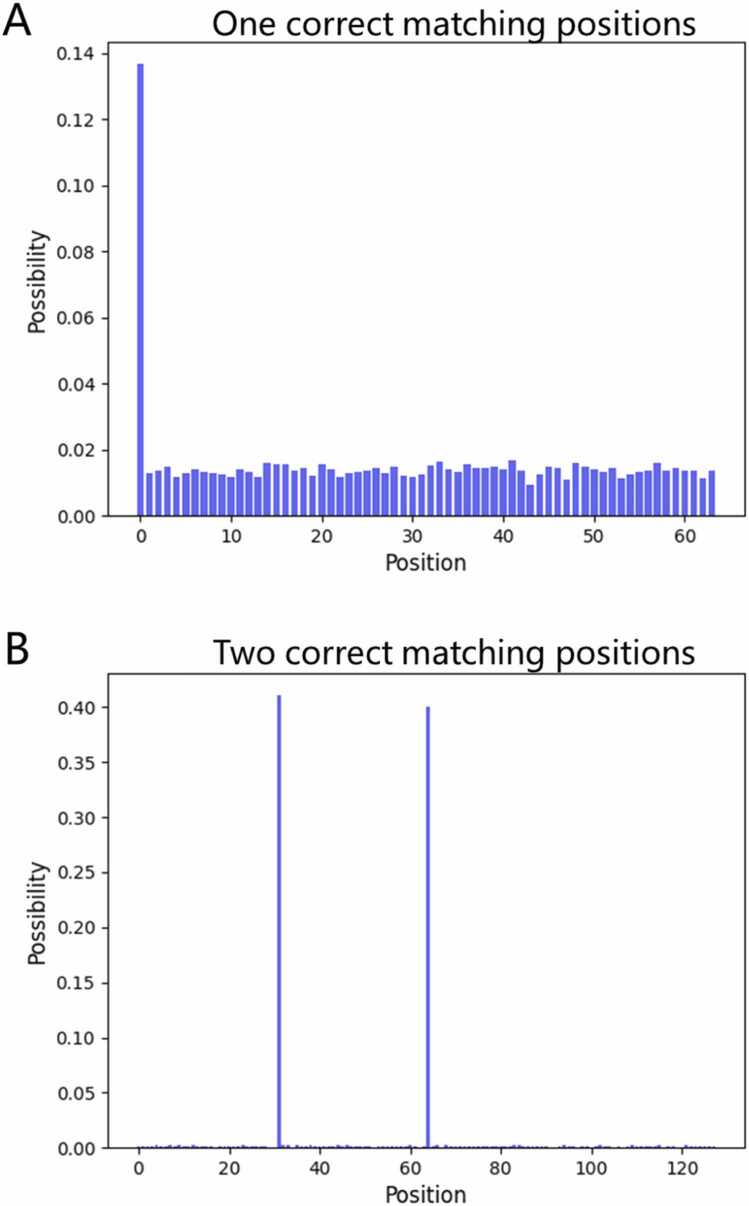
Experiment results on larger cases. (A) Experiment one. (B) Experiment two. The X axis presents the position and the y axis presents the calculated possibilities of the corresponding positions.

To verify its usability on longer sequences, we designed two experiments. For Experiment One, the target sequence is “ccaaaggtaattctaacctgatgcttctggatgtaaatgttttctaaaaaatcatataaatcta”, the pattern sequence is “ccaaaggta” and the correct matching position is the first position. Limited by the hardware limitation, we only repeat the algorithm once, but if we can repeat it more times, the probability of correct matching position can be further amplified. The experiment result of experiment one is shown in Fig. 7A.

For Experiment two, the target sequence is “ccaaaggtaattctaacctgatgcttctggatgtaaatgttttcta-aaaaatcatataaatctatgtatttgattgtttttgacccaaaccgtttacagagccccgggttctgctgcaccgttgggat”, the pattern sequence is “tgta” and the correct matching position is the 32nd and 65th positions. Here, we repeated the algorithm four times and amplified the probabilities of the two correct positions. The result of experiment two is shown in Fig. 7B. As shown in the figure, for both experiments, the probabilities of the correct positions are the greatest, which indicate that the optimized QGSA algorithm can successfully amplify the probability of the correct matching position, and then it validates the usability of our algorithm.

### 3.3 Visualization and analysis websites

**Fig. 8:**
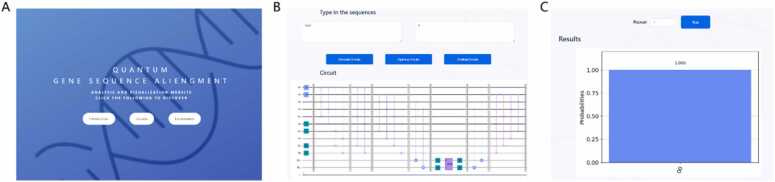
Part of the interface and content of the website. (A) Main page. (B) Interface for generating, optimizing and downloading the circuits. (C) Interface for running the circuits.

To answer the third scientific question, we propose a visualization and analysis website, which provides online circuit generation and simple experiment running. The main functions are detailed in Section 2.3. Most of the interface and content of the website are shown in Fig. 8, and the website is http://www.combio-lezhang.online/QGSA.

As shown in Fig. 8, the users can customize the sequences and the number of repetitions of the circuits online, and then obtain the quantum circuits and results (running on a virtual quantum machine) online. Detailed information and the usage guidance about the websites can be found in the Supplementary Material M3.

## 4 Discussion and conclusion

To answer the three scientific questions mentioned above, this study develops a quantum computing model for sequence annotation of interferon proteins, the corresponding contributions of this article are described as follows: (1) we implement quantum string matching on the basis of the Grover algorithm to construct a quantum sequence alignment algorithm; (2) we propose a quantum circuit optimization algorithm based on the truth table; and (3) we propose a visualization and analysis website.

To answer our first scientific question, we propose a quantum gene sequence alignment algorithm that optimizes the time complexity from linear to semilinear, by combining Grover's quantum search algorithm and the quantum 01 string alignment algorithm. To validate the quantum gene sequence alignment algorithm, we conducted both virtual and real quantum experiments (Fig. 3). The results of the virtual quantum experiment demonstrate the effectiveness of the QGSA circuit, but the results of the expected observation in the real quantum experiment are nonsignificant because of the errors introduced by the large number of gates, resulting in the need to optimize the quantum circuit of the QGSA algorithm, which is our second scientific question.

To answer our second scientific question, we propose the OPTIQUE algorithm to optimize quantum circuits by removing qubits that are not related to the sequence alignment and reconstructing the circuit. To prove the effectiveness of OPTIQUE, Fig. 4 compares the quantum circuits before and after the optimization, which reveals that not only the optimized quantum circuit uses fewer qubits but also the number of quantum gates used by the quantum circuit in the example is reduced. Additionally, we conduct both virtual (Fig. 5) and real (Fig. 6) quantum experiments. On the one hand, the results of the virtual experiment demonstrate the equivalence of the circuits before and after the optimization. On the other hand, the results of the real experiment demonstrate that the errors are obviously reduced and that the probability of observing the correct position is statistically significantly greater than that before optimization (Fig. 6), which validates the effectiveness of our proposed OPTIQUE algorithm.

Notably, although the complexity of the QGSA is reduced to $O(\sqrt{N}{\log_{2}N}^{2})$, the time complexity of the OPTIQUE method is O(N), which makes the optimization of the QGSA algorithm useless. Since each run of the QGSA algorithm requires a run for OPTIQUE, the overall time complexity is O(N) again. This concern is correct for a single run, but our optimizations are meaningful for our interferon gene sequence alignment. In the interferon gene sequence alignment task, the target string corresponds to the gene sequence we need to annotate, and the pattern string corresponds to a known interferon gene pattern. Since the optimized circuits of the target qubits are related only to the target string, we run OPTIQUE once to obtain the optimized circuits. After that, we compile pattern circuits according to different pattern strings in various annotation tasks, which implies that we only need to run OPTIQUE once and then conduct multiple experiments with different pattern strings for a fixed target string. Moreover, since we consider the optimization of the target circuits to be separated from the QGSA algorithm, it can be regarded as preprocessing. Therefore, the optimization of the time complexity from linear to semilinear is valid.

To answer our third scientific question, we construct a visualization and analysis website with four main functions, namely, generating circuits, optimizing circuits, downloading circuits, and running circuits, as shown in Fig. 8.

Although this study has made good progress, several shortcomings still exist. For example, although our proposed QGSA and OPTIQUE algorithms can reduce the time complexity from linear to semi linear, how to successfully and efficiently implement them in actual quantum devices, as well as integrate them into BLAST remains a major challenge.

The scale of the algorithm is also important to discuss. For a target sequence of length N, the space complexity of the QGSA algorithm is $O(N+M+\log_{2}N)$, which is obviously unrealistic for long sequences in quantum computers. Optimized by OPTIQUE, the space complexity is $O(M+\log_{2}N)$, which makes it possible to apply the algorithm. Even for long target sequences, $\log_{2}N$ can be controlled to dozens of quantum bits (If N is 100 million, for example, $\log_{2}N$ is approximately 26), and the demand for the number of quantum bits depends mainly on the pattern sequence length M; in general, we need three qubits to encode a gene in the pattern sequence. Although the existing NISQ quantum computers provide dozens to hundreds of qubits to use [50], the length of the pattern sequence that can be processed is still within this range in theory. Notably, although the space complexity of the optimized algorithm is significantly reduced, longer target sequences can theoretically be queried. However, very long sequences introduce more quantum gates, thereby introducing more errors and making the final result unusable.

Since the gate precision in quantum devices and the error correction of quantum bits are two important issues to be addressed before deployment algorithms to quantum devices, quantum error correction or fault-tolerant quantum computation becomes an important research topic for quantum computing [51]. Many studies have made significant contributions in areas such as quantum fault tolerance and logical qubit encoding [52], [53], [54]. Although current fault-tolerant quantum computing methods and devices cannot support large-scale practical applications [55] and usable quantum fault-tolerant computers request further progress in both algorithms and hardware [51], we still can analyse its potential for speedup within the fault-tolerance regime. As mentioned earlier, our proposed algorithm already optimized the second step of the BLAST algorithm, thereby optimizing the time complexity of the BLAST algorithm. Moreover, once the fault-tolerant quantum computing problem is solved, the depth of the quantum circuit and the accuracy of the quantum gates will not be issues, which indicates our algorithm can be applied to practical problems, thereby optimizing and enhancing the computing efficiency and shortening the computing time in the annotation and DNA sequencing tasks.

In summary, this study proposed a quantum computing model for sequence annotation of interferon proteins, which not only reduces the time complexity of gene sequence alignment from linear to semilinear and provides a useful quantum circuit optimization algorithm, but also leads to the development of a website for online visualization and analysis.

## Author contributions

LZ, BL and YCY conceived the study and developed the model. LZ, BL and YCY performed the simulations for the model and wrote the manuscript. LZ, BL, YCY, FBM and ZYD performed the analysis of the model. All the authors read and approved the final manuscript.

## Avail ability

The code used in this study can be found in https://github.com/skytea/QGSA. The website is http://www.combio-lezhang.online/QGSA.

## Funding

This work was supported by the Noncommunicable Chronic Diseases-National Science and Technology Major Project (No. 2024ZD0532900), the 10.13039/501100001809National Natural Science Foundation of China (No. 62372316), the Sichuan Science and Technology Program Key Project (Nos. 2024YFHZ0091, 2025YFHZ0066).

## CRediT authorship contribution statement

**Bing Li:** Writing – review & editing, Writing – original draft, Visualization, Methodology. **Yangchao Yu:** Writing – review & editing, Methodology, Data curation. **Le Zhang:** Writing – review & editing, Writing – original draft, Methodology, Funding acquisition. **Fubo Ma:** Writing – review & editing, Visualization. **ZiYang Dong:** Writing – review & editing, Visualization.

## Declaration of Competing Interest

none
