# Supplementary material for: Developing a quantum computing model for sequence annotation of interferon protein
Source: Comput Struct Biotechnol J. 2025 Nov 14;30:134–43. doi: 10.1016/j.csbj.2025.11.027 (PMC12664811; doi:10.1016/j.csbj.2025.11.027)
Supplement: Supplementary file 1 — Supplementary material [file mmc1.docx]

**Supplementary Material**

**SUPPLEMENTARY INFORMATION**

**Supplementary Methods**

**Method S1.** Experimental details for quantum circuits

**Method S2.** Example for OPTIQUE algorithm

**Method S3.** Details for visualization and analysis websites

**Supplementary Tables**

**Table S1.** P values of T test between the possibilities of target state “00” and other non-target states of real quantum computer experiment for QGSA

**Supplementary Figures**

**Figure S1.** Xiaohong Quantum Computer Topology

**Figure S2.** Changes in the counting table and the circuits of the index and $t_{0}$ qubits.

**Figure S3.** Normality Test for the experimental data used in Figure 6

**Supplementary Methods**

**Method S1. Experimental details for quantum circuits**

This study employed a virtual quantum computer and a real physical quantum computer to carry out experiments. To ensure the correct results, we repeated each experiment 50 times and calculated the mean and variance as the results. The virtual quantum computer Qasm_simulator is provided by IBM. The key parameter shots (the number of repeated measurements per quantum circuit run) of Qasm_simulator is 5000.

This study used the Snaphong quantum computer provided by the Chinese Academy of Sciences' Institute of Quantum Information and Quantum Technology Innovation for real quantum experiments. Its topology is shown in Figure S1. The red qubit in Figure S1 indicates that the qubit can be used normally, and the gray qubit indicates that the qubit cannot be used.


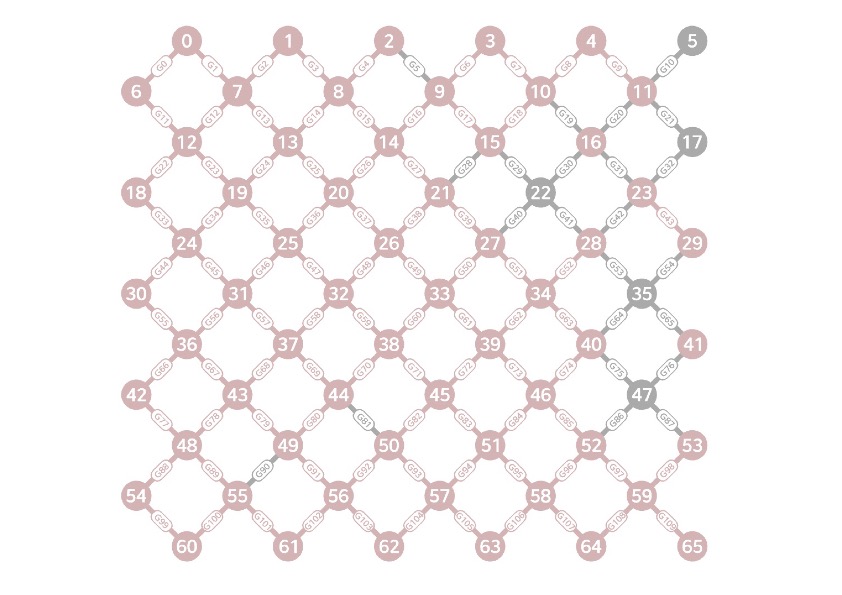


Figure S1. Xiaohong Quantum Computer Topology

The use of Snaphong quantum computer of the study is based on the "ezQpy" package provided by CSHIELD Quantum Computing Cloud platform [1]. "ezQpy" package is a code package based on python, which provides functions such as connecting Snaphong quantum computer for building quantum circuits, running quantum circuits, and obtaining line operation results [1]. More details for individual qubits and quantum gate can be found in the quantum computing cloud platform (https://quantumcomputer.ac.cn) [1]. In the experiment, the mapping between the logical circuits and the physical circuits in the Snaphong quantum computer was implemented by “transpile” function of QuantumCircuit in the Qiskit package [2], and the mapping algorithm was “Sabre” [3].

**Method S2. Example for OPTIQUE algorithm**

In order to present the algorithm clearer, we take the target sequence ACGT (N=4) and pattern sequence A (M=1) as examples for a more intuitive understanding. The target qubit $t_{0}$ is taken as an example to show the process of the fourth step. In Figure S2, A, B, C, and D represent the changes in ${st}_{0}$, the counting table, and the quantum circuit when indices 0, 1, 2, and 3 are iterated, respectively. Here, “a” represents the modified simplified truth table ${st}_{0}$, “b” represents the counting table of $t_{0}$, and “c” represents the quantum circuit. In Figure S2C, the optimization subroutine adds a CNOT gate, and the counting table of $t_{0}$ is updated. Currently, index 2 is iterated, and $IQS_{2}$ (which is 10) has a qubit of 1. In accordance with rule 2, all the positions indicated by the pointer starting from column 1 and row 3 of the counting table are added.

Figure S2. Changes in the counting table and the circuits of the index and $t_{0}$ qubits. Here, IB, TB and QC represent the index qubit, target qubit and quantum circuit, respectively.

**Method S3. Details for** **visualization and analysis websites**

Here, we present a detailed introduction for the functions and usages of the visualization and analysis website. Four functions are provided on the website:

First, users can generate quantum circuits corresponding to the QGSA. Users can input any legal gene sequence as the search target sequence and then input the pattern sequence. The website generates quantum circuits corresponding to the input sequences, which are subsequently displayed as figures.

Second, users can generate an optimized quantum circuit. After the quantum circuit corresponding to the QGSA is generated, users can select the quantum circuit to optimize. The website optimizes the quantum circuit according to the OPTIQUE optimization algorithm and displays the optimized quantum circuit.

Third, users can download the quantum circuit after generation. Users can download quantum circuits in QASM format [2].

Fourth, users can run quantum circuits. After the circuit is generated and optimized, the users can customize the run times for the quantum gene sequence alignment algorithm and run the quantum circuit on the virtual quantum computer. The website displays the results of the proposed circuits, which correspond to the location of the pattern sequence in the target sequence.

For the usages, there are some guides:

As shown in Figure 8A, users can directly click on the corresponding “Introduction”, “Circuits”, and “Experiments” buttons of the home page, and subsequently go to the introduction, circuit generation, and experiment sections.

As shown in Figure 8B, users can input the target sequence and the pattern sequence in the two input text boxes. After that, by clicking the "Generate Circuits" button, the corresponding quantum circuit can be generated. The “Optimize Circuits” button enables the execution of the optimization algorithm and generates the optimized quantum circuit. The "Download Circuits" button allows the corresponding quantum circuit to be downloaded.

As shown in Figure 8C, users can enter numbers in the text box corresponding to the number of iterations of the algorithm. After users click “Run” to execute the circuits online, the results are displayed below.

**Supplementary Tables**

**Table S1**

Supplementary Table S1. P values of T test between the possibilities of target state “00” and other non-target states of real quantum computer experiment for QGSA

| Pairs | P value |
| --- | --- |
| “00” - “01” | 0.804 |
| “00” - “10” | 0.902 |
| “00” - “11” | 0.918 |

**Supplementary Figures**

**Figure S3**

Supplementary Figure S3. Normality Test for the experimental data used in Figure 6. The number on the figure is the P value calculated by Shapiro-Wilk test. All the P values are bigger than 0.05, indicating that all the experimental data follow a normal distribution.


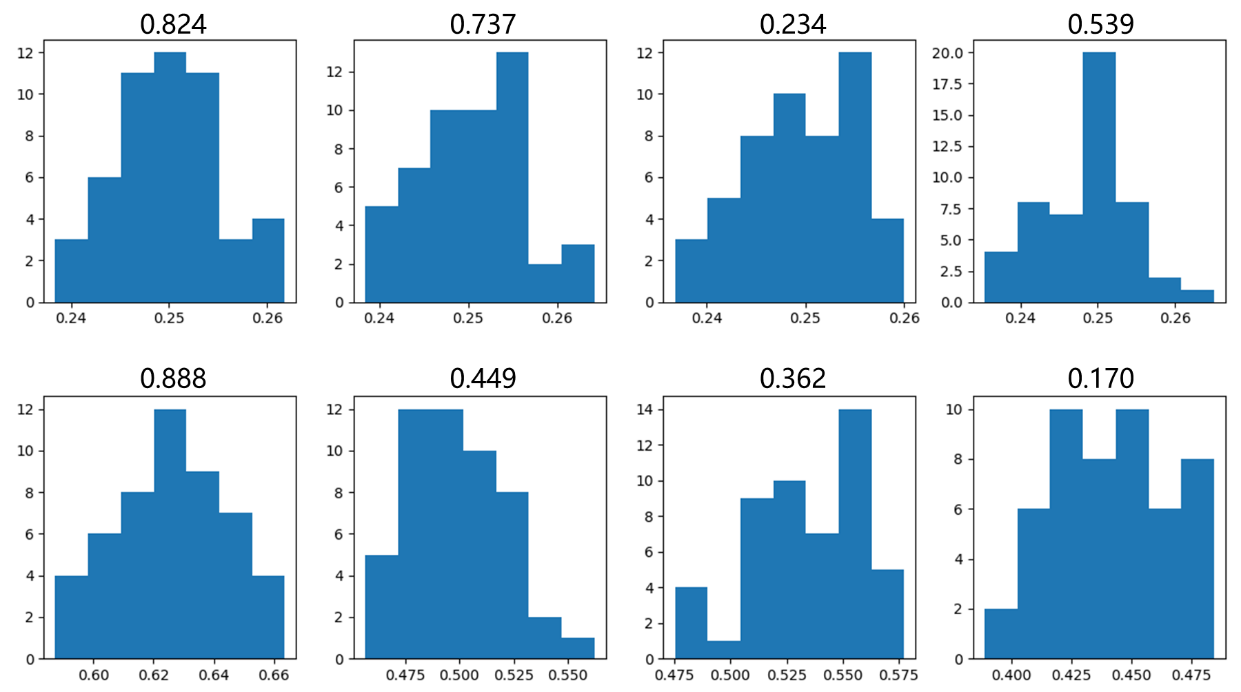


**Reference**

[1] PHYSICS, C. C. F. E. I. Q. I. A. Q., 2017.

[2] Javadi-Abhari, A., Treinish, M., Krsulich, K., Wood, C. J.*, et al.*, Quantum computing with Qiskit. 2024, *abs/2405.08810*.

[3] Li, G., Ding, Y., Xie, Y., Tackling the Qubit Mapping Problem for NISQ-Era Quantum Devices. *Asplos '19* 2019, 1001–1014.
